# Why Are Young Adults Affected? Estimating Measles Vaccination Coverage in 20-34 Year Old Germans in Order to Verify Progress Towards Measles Elimination

**DOI:** 10.1371/currents.outbreaks.0a2d3e9465f067a0b2933d598d504d2e

**Published:** 2015-02-25

**Authors:** Melanie Schuster, Thomas Stelzer, Florian Burckhardt

**Affiliations:** Postgraduate Training for Applied Epidemiology (PAE), Robert Koch Institute, Berlin, Germany; Landesuntersuchungsamt Rheinland Pfalz, Germany; Landesuntersuchungsamt Rheinland Pfalz, Germany; Landesuntersuchungsamt Rheinland Pfalz, Germany

**Keywords:** vaccine hesitancy

## Abstract

Background: The introduction of measles vaccination into routine childhood vaccination programmes has led to a shift of disease burden and incidence among young adults. This was confirmed by the recent rise in measles cases and outbreaks throughout Europe. To prevent outbreaks and eliminate measles, one of the key objectives of the WHO Europe measles elimination framework is achieving overall vaccination coverage of ≥95% in the population on a district level. In the absence of national registers, data on vaccination coverage in Germany is recorded at the age of school entry, through insurance refund claim data and population studies. Vaccination status (VS) of young adults is largely unknown. Methods: We assessed measles vaccination coverage in young adults aged 20-34 years on a district level of the German Federal State of Rhineland-Palatinate. The knowledge and attitude towards immunization of unvaccinated to vaccinated young adults were compared using Likert questions. We used proportional allocation for stratified random sampling across 36 counties. We mailed a self-administered questionnaire with pre-paid return envelopes along with an offer to complete online. Prior to calculating coverage we tested for non-responder bias using logistic regression. Results: 465 (28%) of 1,637 persons contacted responded (mail: 23%, online: 5%). More women responded than men (odds ratio (OR)=2.1; 95% confidence intervall (CI)=1.7-2.6) but age did not vary between responders and non-responders. Vaccination coverage was 90% (95%CI=87%-93%) for one and 56% (95%CI=51%-61%) for two doses. We found a statistically significant association between receiving two doses and age group. The 20-24 years age group had a 2.3 higher incidence rate ratio (95%CI=1.7-3.2) than the reference group of 30-34 year old to have received two doses of measles vaccination. The group of 25-29 year old had a 1.5 higher incidence rate (95%CI=1.0-2.1) than the reference group to have received two doses of measles vaccination. Conclusions: Coverage has failed to reach the WHO Europe elimination goal of 95% measles vaccination in the general population. Targeted approaches including enlistment of occupational health services and checking vaccination status during general practitioner (GP) visits are needed to increase vaccination uptake in this age group in order to achieve measles elimination.

## Related Articles

The article is part of the *PLOS Currents Outbreaks *"Vaccine Hesitancy Collection".

## Introduction

Measles are a highly communicable viral disease manifesting with a characteristic maculopapular rash beginning on the third to seventh day after infection accompanied by cough, coryza and/or conjunctivitis. Severe cases can lead to pneumonia, meningitis, subacute sclerosing panencephalitis or death. Disease severity increases with age. No causal therapy is available; vaccination serves as primary preventive measure.

Europe has been struggling to fight measles in the last decades and has so far failed to achieve the goal of eliminating the disease. The year 2015 has been set as a new target for the elimination of measles in the European region. Yet in 2011 the number of measles cases were again rising throughout Europe, with 36 out of 53 Member States in the WHO European Region reporting outbreaks, noting >26 000 cases in 2011. In France 5090 cases were being claimed in 2010 and more than 14000 cases in 2011. 1 ^,^ 2

From April 2012 to March 2013 a total of 8127 cases were reported from 30 contributing EU and EEA member states. During this 12 months period Germany, France, Italy, Romania, Spain and the United Kingdom accounted for 95% of the cases.^3^

Measles are notifiable by law in Germany. The elimination of measles in Germany has progressed after introduction of vaccination thirty years ago, yet measles still pose a threat to the German public health with large-scale-outbreaks of up to several hundred cases in the recent years. The largest documented outbreak in the previous years was reported in 2006 with a number of 1452 cases.^4^ Further outbreaks were reported the same year, one being linked to an anthroposophic school.^5^^,^ 6 Also linked to an anthroposophic school were two outbreaks 2010.^7^^,^ 8 Apart from national outbreaks, measles were exported from Germany to Bulgaria causing a large-scale outbreak within a Roma population.^9^ Reasons for infection in a high-income country such as Germany are mostly missing immunization, philosophical objection against vaccination (e.g. anthroposophic believes), or travel-related from countries with a high number of measles cases. Philosophical objectors represent 3-5% of the German population^10^ yet only 1% of German parents indicate total refusal towards vaccination.^11^

In the absence of national registers, standardized data on vaccination coverage in Germany is only obtained at the age of school entry, which is normally at the age of six. Coverage for measles (one dose) is consistently above 90%. However, introduction of measles vaccination into routine childhood immunization has shifted the pool of susceptibles to older age cohorts who have neither received vaccination nor obtained natural immunity through infection.

The outbreaks registered in Europe in the last years confirm an increase in cases among young adults who have had no previous vaccination against measles. Half of the cases in 2011 in Europe occurred among people aged ≥15 years, reflecting in a high proportion of adult cases in Germany.^12^ This age cohort of young adults is at higher risk of infection and at higher risk of developing severe disease. At child-bearing age, this age-group could further pass on the disease to infants (<11month) for whom vaccination is not recommended.

Due to the rise in cases in adults, the German Standing Committee on Vaccination (STIKO) extended the eligible age-group for measles vaccination in 2010. One dose of measles containing vaccine is recommended for unvaccinated adults or adults having received only one dose and born after 1970.^13^

Data on vaccination coverage in adults are missing in Germany and progress towards the WHO elimination goal cannot be verified adequately, neither can health promotion campaigns be tailored to people-at-risk.

We therefore conducted a representative cross-sectional survey among 20-34 year old young adults to assess the vaccination status of this age-group in Rhineland-Palatinate (RP), a federal state in the Southwest of Germany. This will enable verification towards the measles elimination goal and if necessary allow targeted measures to increase coverage.

## Methods

The total population of RP is 4 Mio inhabitants^14^, with 1,3 Mio between the age of 20 and 34. Germans are required by law to be registered at place of residence and our sampling frame were the population registries of the counties of RP.

We used proportional allocation^15^ to calculate sample size and sampled randomly, stratified across the 36 counties to assure representativeness. Our sampling parameters were: an expected vaccination coverage of 50% (conservative assumption of expected vaccination coverage), precision of 10% and a 95% CI. This yielded a sample size of 385 which together with our expected response rate of 20% required 1925 addresses from the local county registries.

They were asked to randomly draw a number of participants allotted to their county, distributed equally between sex and age. Postal address, gender and age were submitted to the study team.

Participants were contacted by post. We mailed a self-administered pseudonymous paper questionnaire along with instructions how to complete an online form of the same questionnaire. Participants were therefore given the choice to either return the paper form or access an online-based questionnaire. An access token was included in the primary posting in order to mark multiple online entries.

Several means were used to increase overall response rate. Pre-paid return envelopes as well as an incentive, a small pen, was included in the posting. Additionally, a reminder letter was sent out if there was no response to the primary letter within 30 days of initial contact.^16^

The questionnaire contained demographic characteristics (sex, age, country of birth and district). Vaccination status and source of vaccination status were ascertained. Likert questions were used to assess the level of agreement or disagreement on statements in relation to the protection conferred by immunization, the information policy regarding adverse-events following immunization and the knowledge concerning the immunization recommendation for young adults in Germany introduced in 2010.

Data was analysed using Stata 11. 95% confidence intervals were calculated using multivariate logistic or poisson regression with a p-value of <0.05 being considered significant for all statistical tests. Overall vaccination coverage for at least one and for two doses were calculated using binomial exact methods. To ensure the representativeness of the sample, a non-responder analysis was conducted comparing age and sex of those who had responded to those who had not responded (obtained by local council registries) using multivariate poisson regression with response as dependent variable.

Multivariate logistic regression was used to compare online with paper response as dependent variable (mode of reply) to examine the effect of vaccination status, age group and sex.

We also tested the effect of age group and sex on vaccination status using multivariate logistic regression. Respondents with missing source of vaccination status or unknown or missing vaccination status were excluded from analyses on vaccination coverage. Odds ratios were calculated to compare the knowledge and attitude towards immunization of unvaccinated to vaccinated young adults.

The study protocol was approved by Medical Doctors Ethics Committee of Rhineland-Palatinate as well as the privacy officer of RP. Participants were advised that informed consent was given by completing and returning the questionnaire. Current data protection law was taken into account.

## Results

From June 2012 to August 2012, a representative sample of 1,637 persons, equally distributed between gender and age, were contacted. Overall response rate was 28% (n=465). 23% (n=387) of the persons contacted responded by mail, 5% (n=78) used the online version of the questionnaire to reply. 63% of the participants were female (n=293), 37% male (n=169) and three gave no information on their gender. Data were complemented for the analysis using the list provided by the population registries. (Figure 1)

**Responders:**
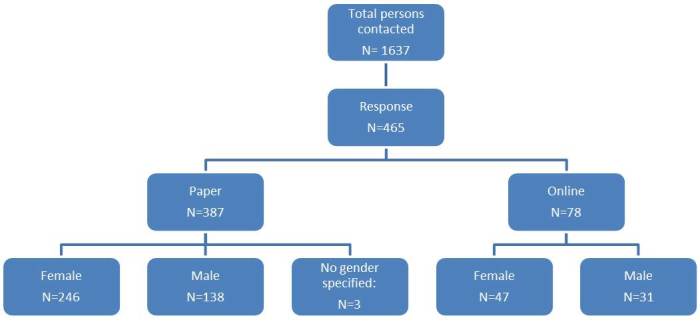

Mode of reply (online vs. paper) did not vary by age group (20-24: OR=0.9; p=0.8; 95%CI=0.5-1.8; 25-29: OR=1.3; p=0.4; 95%CI=0.7-2.4; 30-34: reference group) or sex (OR=1.1; p=0.7; 95%CI: 0.6-1.8). There was no difference of vaccination status between online and paper participants: no vaccination (OR=0.8; p=0.7; 95%CI=0.3-2.2), one vaccination (OR=1.2; p=0.5; 95%CI: 0.7-2.3) or two vaccinations (reference group). The only difference we found was that online respondents were less likely to know their vaccination status (OR=3.5; p=0.002; 95%CI=1.6-7.8).

Age did not vary significantly among responders and non-responders (20-24: odds ratio (OR) = 0.8; p= 0.2; 95%CI= 0.6-1.1; 25-29:OR= 0.9; p= 0.3; 95%CI:0.7-1.1; 30-34: reference), yet more women responded than men (OR=2.1; 95%CI=1.7-2.6).

455 out of all 465 responders answered the question on source of vaccination status. Of those, 80% (n=362) obtained the information from their vaccination card and 2% (n=8) had consulted their GP. (Table 1)

**Table 1: Source of information on vaccination status, if known (n=455):** 

Source of information	Total number (in %)
Vaccination card	362 (80%)
Memory	57 (12%)
Parents	25 (5%)
GP	8 (8%)
Other	3 (1%)

Of the total of 455 participants who provided the source of vaccination status, we excluded those participants with missing or unknown vaccination status leaving 415 participants for the analysis on vaccination status. (Figure 2)

**Participants for analysis:**
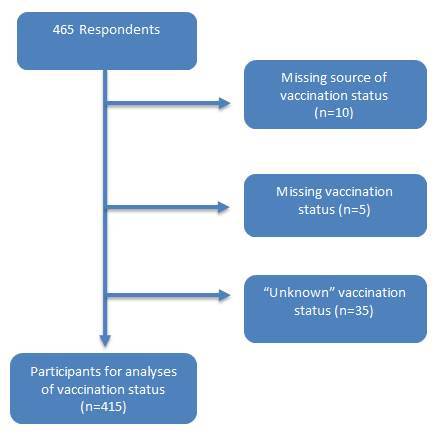


Vaccination coverage was 90% (95%CI=86%-92%) for one or more doses and 56% (95%CI=51%-61%) for two doses. 10% (95%CI=7%-13%) of the study population was unvaccinated. (Table 2)

**Table 2: Vaccination status of the study population:** 

Vaccination status	Total number (n)	Percentage (%)	95% CI
Measles vaccination (1x or more)	374	90%	86%-92%
Measles vaccination (2x)	234	56%	51%-60%
Unvaccinated	41	10%	7%-13%
		100%	

Distribution of vaccination status by sex and age-group identified a higher crude number of vaccinated women than men. (Table 3)

**Table 3: Vaccination status (one or more doses) by age-group and sex:** 

Vaccination status	Sex	20-24 years Total number (n)	20-24 years Percentage uptake (%)	25-29 years Total number (n)	25-29 years Percentage uptake (%)	30-34 years Total number (n)	30-34 years Percentage uptake (%)
Measles vaccination (1x or more)	Male	55	96	29	83	41	79
	Female	87	99	78	88	84	89
Measles vaccination (2x)	Male	46	96	12	67	18	62
	Female	72	99	51	88	35	71
Unvaccinated	Male	2	0	6	0	11	0
	Female	1	0	7	0	14	0

In poisson regression we did not find differences in vaccination status between the sexes for either "at least one" or two doses of measles vaccination. There was no statistical difference between the age groups for "at least one vaccination" (results not shown).

However, we found a statistically significant association between receiving two doses and age group. The 20-24 years age group had a 2.3 higher incidence rate ratio (95%CI=1.7-3.2) than the reference group of 30-34 year old to have received two doses of measles vaccination. The group of 25-29 year old had a 1.5 higher incidence rate (95%CI=1.0-2.1) than the reference group to have received two doses of measles vaccination. (Table 4)

**Table 4: Poisson regression of age group and having received two doses of measles vaccination:** 

**Receiving two doses of measles vaccination**	**Incidence rate ratio**	**p-value**	**95% CI**
20-24 years	2.3	<0.001	1.7-3.2
25-29 years	1.5	0.037	1.0-2.1
30-34 years	reference		
Sex (male= reference)	1.1	0.328	0.9-1.5

Within the group of the unvaccinated, 85% agreed or strongly agreed that vaccines induce effective protection against infectious diseases, 5% disagreed that immunizations protects against infectious diseases. No significant difference to the group of the vaccinated could be observed. Nevertheless 73% of the unimmunized indicated that they agreed or strongly agreed with the statement that the general public was not informed well enough on adverse events following immunization (AEFIs). 20% disagreed or strongly disagreed that the general public was not well informed on AEFIs. No significant difference to the group of the vaccinated could be observed.

46% of the unimmunized indicated that they had no opinion on the issue whether unvaccinated adults or adults having received only one dose of measles containing vaccine should be immunized against measles.

23% answered that they disagreed or strongly disagreed that unvaccinated adults should receive measles immunization. 31% indicated that they agreed or strongly agreed that unvaccinated or one-dose-vaccinated adults should receive measles immunization. Agreement with the statement was associated with a statistically significant four times higher OR of being vaccinated. (Table 5)

**Table 5: Univariate logistic regression of Likert response on having received at least one dose of measles vaccination:** 

Likert question	Number of vaccinated (n=374)	Number of not vaccinated (n=41)
**Immunization induces effective protection against infectious diseases. **		
Agree or strongly agree	344	34
Do not "Agree or strongly agree"*	27	6
Total respondents	371 (3 missing)	40 (1 missing)
Odds ratio: 2.3	p-value. 0.234	95%CI: 0.7-6.1
**The general public is not informed well enough on AEFIs. **		
Agree or strongly agree	238	30
Do not "Agree or strongly agree"*	35	11
Total respondents	373 (1 missing)	41
Odds ratio: 0.7	p-value: 0.234	95%CI: 0.3-1.4
**Unvaccinated adults or adults having received only one dose of measles containing vaccine should be immunized against measles. **		
Agree or strongly agree	239	12
Do not "Agree or strongly agree"*	9	27
Total respondents	373 (1 missing)	39 (2 missing)
Odds ratio: 4.0	p-value: <0.001	95%CI: 1.9-9.0
* includes "no opinion"		

## Discussion

We minimized sampling bias by providing detailed instructions to population registries. To avoid study participants from providing deviant answers the questionnaire was developed being non-judgmental and data-protection issues were stressed in the cover letter. Nevertheless, a questionnaire based survey is susceptible to refusal bias especially by philosophical objectors. The latter are generally un- or undervaccinated. A refusal to participate would lead to overestimated vaccination coverage. Philosophical objectors are however a minority in the German population (3-5%) and their effect on overall coverage in this survey is negligible. A response bias by providing counterfactual answers could have occurred in case participants were reluctant to confide secrets or for reasons of social desirability.

Beyond a possible refusal of philosophical objectors to respond, there might be a selection bias towards those with a greater health-seeking behaviour, hence a higher tendency for vaccinated individuals to respond to this survey which in return might lead to an overestimation of the vaccination coverage. To encounter this and ensure that a representative sample of the society responds, participation was encouraged by sending an incentive with the questionnaire, a pre-paid return envelope and by sending out a recall letter. In case of a missing vaccination card, recall bias was possible as vaccination against measles might not be remembered correctly. Study participants were encouraged to contact their GP about their vaccination status or contact their parents or legal guardians. 82% of answers were based on either vaccination cards or GPs information.

One fifth of our study population chose to respond online. No difference was found in response between the two groups with regard to age, vaccination status or, interestingly, sex. Frequent use of internet is common among young adults of both sexes and adding online questionnaires should be considered as a cost-saving method when addressing this age-group to increase response in surveys.

Our results show that unlike today, where measles vaccination coverage for two doses at school entry is assessed to be 92,1%,^17^ measles vaccination recommendations were not sufficiently implemented during the childhood of the investigated age-groups. This is especially true for the 30-34 year age group whose members are more than two times less likely to have received two doses of measles vaccination compared to the 20-24 year olds. These findings were confirmed by a recent survey which also reports that measles vaccination coverage in Germany is higher in younger age groups.^18^Reasons might be high wild-type virus circulation, decreased acceptance of vaccination or missing knowledge of recommendations.

In addition, measles might be considered as a harmless childhood disease by the general public, and possibly even by paediatricians. The majority of parents of our target age group were highly likely to have had measles during childhood. Measles are more severe among young adults. Disease in this age group was not seen frequently in the past as the greatest disease burden was within children; hence parents might have deemed vaccination of their children as unnecessary.

Our survey showed that 90% of adults between 20 and 34 were vaccinated at least once and 56% received two doses of measles vaccine. The WHO Europe measles elimination framework recommends vaccination coverage of ≥95% with two doses of measles-containing vaccine at the subnational administrative level to interrupt virus transmission.^19^ In order to reach that goal, targeted approaches are needed to promote vaccination for these identified age-groups. The results of our study indicate that the majority both unimmunized and immunized are confident concerning the protection induced by immunization, though the majority also agreed that the general public was not informed well about AEFIs, possibly leading to skepticism towards immunization. Almost half of the unimmunized in our study had no opinion on measles vaccination for adults; one out of four indicated that measles vaccination was not needed as an unvaccinated adult. The knowledge on this differed significantly between unvaccinated and vaccinated individuals, which might indicate a lack of awareness of the recommendations on measles immunization for adults introduced in Germany in 2010. Every contact with health services should be used to check patient’s vaccination status, inform all patients about the actual risk of adverse events following immunization and offer measles immunization to unvaccinated or one-time-only-vaccinated young adults. Awareness should be raised among physicians, especially GPs, gynaecologists and paediatricians in contact with parents visiting the health facility to get their child vaccinated, as they are presumably are the most likely to be in contact with this age-group. The adult population at work could be approached by their occupational health services to check up on their measles vaccination status and thus avoid loss of work.

## Correspondence

Melanie Schuster

Email: Schusterm@who.int
